# Globular Adiponectin Causes Tolerance to LPS-Induced TNF-α Expression via Autophagy Induction in RAW 264.7 Macrophages: Involvement of SIRT1/FoxO3A Axis

**DOI:** 10.1371/journal.pone.0124636

**Published:** 2015-05-11

**Authors:** Nirmala Tilija Pun, Amit Subedi, Mi Jin Kim, Pil-Hoon Park

**Affiliations:** College of Pharmacy, Yeungnam University, Gyeongsan, Gyeongsangbuk-do 712–749, Republic of Korea; Northwestern University Feinberg School of Medicine, UNITED STATES

## Abstract

Adiponectin, an adipokine predominantly produced from adipose tissue, exhibited potent anti-inflammatory properties. In particular, it inhibits production of pro-inflammatory cytokines, including tumor necrosis factor-α (TNF-α), in macrophages. Autophagy, an intracellular self-digestion process, has been recently shown to regulate inflammatory responses. In the present study, we investigated the role of autophagy induction in the suppression of Lipopolysaccharide (LPS) -induced TNF-α expression by globular adiponectin (gAcrp) and its potential mechanisms. Herein, we found that gAcrp treatment increased expression of genes related with autophagy, including Atg5 and microtubule-associated protein light chain (LC3B), induced autophagosome formation and autophagy flux in RAW 264.7 macrophages. Similar results were observed in primary macrophages isolated peritoneum of mice. Interestingly, inhibition of autophagy by pretreatment with Bafilomycin A1 or knocking down of LC3B gene restored suppression of TNF-α expression, tumor necrosis factor receptor- associated factor 6 (TRAF6) expression and p38MAPK phosphorylation by gAcrp, implying a critical role of autophagy induction in the development of tolerance to LPS-induced TNF-α expression by gAcrp. We also found that knocking-down of FoxO3A, a forkhead box O member of transcription factor, blocked gAcrp-induced expression of LC3II and Atg5. Moreover, gene silencing of Silent information regulator 1 (SIRT1) blocked both gAcrp-induced nuclear translocation of FoxO3A and LC3II expression. Finally, pretreatment with ROS inhibitors, prevented gAcrp-induced SIRT1 expression and further generated inhibitory effects on gAcrp-induced autophagy, indicating a role of ROS production in gAcrp-induced SIRT1 expression and subsequent autophagy induction. Taken together, these findings indicate that globular adiponectin suppresses LPS-induced TNF-α expression, at least in part, via autophagy activation. Furthermore, SIRT1-FoxO3A axis plays a crucial role in gAcrp-induced autophagy in macrophages.

## Introduction

Although adipose tissue was originally recognized as a major site for the storage of excess energy, a growing body of evidence suggested that it acts as an important endocrine organ via secretion of a number of biologically active hormones collectively called “adipokines”. Of the various types of adipokines, adiponectin was reported to play a key role in insulin sensitization and lipid metabolism [1]. Recent clinical evidences have demonstrated that low level of circulating adiponectin is associated with pathophysiology of type 2-diabetes, obesity and cardiovascular disease [2], indicating a beneficial role of adiponectin in the human physiology. In addition, adiponectin also exhibited the potent anti-inflammatory properties by suppressing the production of various inflammatory cytokines, including TNF- α, interleukin 6 and 8, as well as generation of anti-inflammatory signals [3].

It has been reported that long time pretreatment of macrophages with adiponectin suppresses LPS-induced TNF-α expression [4], whereas adiponectin itself rapidly induces expression of inflammatory cytokines [5], indicating that long term effect of adiponectin generates tolerance to TNF-α expression in response to LPS stimulation in macrophages. Tolerance is considered as a protective mechanism to limit the inflammatory damage as a consequence of excessive activation of monocytes and macrophages either via inhibition of signaling involved in inflammatory gene expression, production of anti-inflammatory cytokines or down-regulation of endotoxin receptor [6]. With regards to the generation of tolerance effects by adiponectin, various mechanisms have been proposed. For example, LPS-stimulated production of pro-inflammatory cytokines, such as, TNF-α and IL-6, was terminated by pretreatment with adiponectin via inducing expression of IL-10 and IL-1 receptor antagonist (IL-RA) [7,8]. Adiponectin also induced expression of inactive isoform of IL-1R-associated kinase (IRAK) family of kinases, which is a component of toll-like receptor (TLR) signaling and required for TNF-α expression, via formation of complex with TNF receptor-associated factor 6 (TRAF6) [9]. In addition, long term pretreatment with adiponectin suppressed LPS-induced activation of NF-κB and MAPK signaling in macrophages [3]. Although many previous reports have shown the tolerance effect of adiponectin to inflammatory responses, the detailed molecular mechanisms underlying are still largely unknown.

Autophagy is an intracellular self-digestive process that allows removal of damaged and potentially toxic intracellular components by delivering them into lysosomes [10]. Autophagic process is classified by autophagosome formation, fusion of autophagosome with lysosome (autolysosome formation) and finally degradation of target molecules, each of which is coordinately regulated by a number of genes-related with autophagy (Atgs), such as Beclin-1, Atg12, Atg5 and Atg8 (LC3) [11]. In addition to the essential roles in the regulation of cell survival and/or death, increasing evidence has demonstrated that autophagy is implicated in many other physiological responses, and dysfunction of autophagy is therefore closely associated with various pathophysiological states [12]. For example, recent studies have highlighted a pivotal role of autophagy in the regulation of innate immune system and inflammatory responses. Autophagy has been shown to suppress inflammasome activation in response to omega-3 fatty acid [13] and prevent angiotensin II-induced inflammation in macrophages [14]. On the other hand, autophagy process has also been shown to mediate inflammation in lung [15], pro-inflammatory response depicted by secretion of IL-6, TNF-α and IL-8 [16] and induce inflammasome activation in cells dying through autophagy. Taken together, these results suggest that autophagy would modulate inflammatory responses in a context-dependent manner.

FoxO3A, a member of Forkhead box O (FoxO) transcription factor, has been shown to play a conserved role in maintaining cellular homeostasis via modulation of apoptosis and cell cycle [17]. For example, up-regulation of FoxO3A forces cells to undergo apoptosis [18], which is correlated with long term survival of cancerous patient [19]. In contrast, FoxO3A signaling plays a cytoprotective role against oxidative stress by enhancing expression of anti-oxidant genes [20]. In addition, recent studies have revealed that FoxO3A plays a critical role in autophagy induction in response to stress stimuli via induction of various genes related with autophagy [21]. We have previously reported that adiponectin treatment induces autophagy through activation of FoxO3A, which is implicated in the protection of liver cells from ethanol-induced apoptosis by adiponectin [22], indicating a possibility that FoxO3A would be a critical target in regulation of various biological responses by adiponectin. However, the mechanism underlying the role of FoxO3A signaling in the vicinity of anti-inflammatory response by adiponectin has not been explored yet.

Silent information regulator 1 (SIRT1), a member of class III histone deacetylase (HDAC), generates various biological actions via acting on a number of non-histone targets, including nuclear factor-κB (NF-κB), tumor suppressor p53 [23], which results in alteration of biological activities of target substrates, as well as on histones. It has been shown that SIRT1 expression is enhanced in response to stressful conditions, including food/caloric restriction and oxidative stress [24,25] and produces anti-oxidative and anti-apoptotic effects [23]. Moreover, SIRT1 expression is closely associated with autophagy induction and activation of FoxO3A signaling [26,27]. Taken together, these previous reports strongly suggested the potential role of SIRT1 and FoxO3A in modulation of various biological responses by adiponectin, in particular regulating cell survival/death and oxidative stress. However, the role of SIRT1 signaling in FoxO3A activation by adiponectin in macrophages and further its role in the development of anti-inflammatory responses by adiponectin has not been explored.

Based on previous reports, it is widely accepted that long term treatment of macrophages with adiponectin generates tolerance to LPS-induced TNF-α expression. Although it has been shown that adiponectin modulates various signaling pathways involved in TNF-α production, detailed mechanisms underlying are still largely unknown. Thus, to better understand the molecular mechanisms underlying suppression of LPS-induced inflammatory responses by adiponectin; herein we investigated the role of autophagy induction in the suppression of LPS-induced TNF-α expression. In this study, we have clearly demonstrated the evidence that suppression of LPS-induced TNF-α expression by globular adiponectin is mediated, at least in part, via autophagy induction. In addition, we further explored the potential mechanisms underlying and showed that SIRT1/FoxO3A axis plays a crucial role in autophagy induction by globular adiponectin in RAW 264.7 macrophages.

## Materials and Methods

### Materials

All the cell culture reagents were purchased from Hyclone Laboratories (South Logan, UT, USA). Recombinant human globular adiponectin (gAcrp) was acquired from Peprotech Inc. (Rocky Hill, NJ, USA) and 5-Chloromethyl-2, 7-dichlorodihydrofluorescein diacetate (CMH2DCFDA) from Molecular Probes (Eugene, OR). NADPH, diphenyleneiodonium chloride (DPI) and Lucigenin were purchased from Enzo Life Sciences (Farmingdale, NY, USA). Rabbit polyclonal antibodies against LC3B (Cat.No.2775), Beclin-1 (Cat.No.3738), total p38MAPK (Cat.No.9212) and rabbit monoclonal antibodies against FoxO3A (Cat.No.2497) and phospho-specific p38MAPK (Cat.No.9215) were obtained from Cell Signaling Technology Inc. (Beverly, MA, USA). Rabbit polyclonal antibody against Atg5 (Cat.No.PAI-46178) and rabbit monoclonal antibody against β-actin (Cat.No.04-1116) were purchased from Thermo Scientific Inc (Rockford, IL, USA) and rabbit polyclonal antibodies against TRAF6 (Cat.No.SC-7221), SIRT1 (Cat.No.SC-15402) and Lamin-B1 (Cat.No.20682) were obtained from Santa Cruz (Delaware, CA).

### Cell cultures

RAW 264.7 macrophage cell line (Cat.No.KCLB-40071) was purchased from the Korean cell line bank (Seoul, Korea) and routinely cultured in Dulbecco’s modified Eagle’s medium (DMEM), supplemented with 10% (v/v) Fetal Bovine Serum(FBS) and 1% (v/v) penicillin-streptomycin at 37°C in an incubator with 5% CO2.

### Isolation and subculture of murine peritoneal macrophages

Peritoneal macrophages were isolated from 5- to 7-week old, male BALB/c mice as described previously [28]. Briefly, mice were Intraperitoneal (I.P) injected with 1 ml of 4% (W/V) Brewer thioglycollate medium (Difco, Detroit, MI) to stimulate the accumulation of macrophages in the peritoneum. On third day of injection, peritoneal cells were isolated by washing the peritoneal cavity with 10 ml of ice-cold Hank's balanced salt solution (HBSS)-free of calcium and magnesium. The cells were centrifuged at 12,000 rpm for 5 min and washed with RPMI 1640 containing 10% fetal calf serum (FCS) and 1% penicillin-streptomycin. Cells were then seeded in culture dish with RPMI 1640 containing 10% FCS, 1% penicillin–streptomycin at 37°C in an incubator with a humidified atmosphere of 5% CO2. These cells were used for the further experiments.

### ELISA for TNF-α detection

RAW 264.7 macrophages and murine peritoneal macrophages were seeded at a density of 5× 10^4^ cells/well in 96-well plates and pretreated with Bafilomycin A1 in presence or absence of gAcrp along with LPS or transfected with LC3B siRNA followed by treatment with gAcrp and LPS for the indicated time periods. The culture media were then collected and used to measure the TNF-α protein secreted in the media by using TNF-α ELISA kits (Biolegend, San Diego, CA, USA) according to the manufacturer’s instructions.

### RNA isolation, reverse transcription (RT) and quantitative PCR (qPCR)

For the measurement of mRNA levels of target genes, total RNAs were isolated by Qiagen lysis solution (Qiagen, Maryland, USA) according to the manufacturer's instruction. Isolated total RNA was reverse transcribed into cDNA using Go Script reverse transcription system (Promega). Quantitative Real time-PCR was then performed using Light Cycler 2.0 (Mannheim, Germany) with the use of absolute QPCR SYBR green capillary mix AB gene system (Thermoscientific, UK) at 95°C for 15 s, 56°C for 30 s, and 72°C for 45 s. The amount of target mRNA was analyzed via comparative threshold (Ct) method after normalizing target mRNA Ct values to that for glyceraldehyde-3-phosphate dehydrogenase (GAPDH) (ΔCt). The primer sequences used for amplification of target genes are listed in Table 1.

**Table 1:** Sequences of primer used in quantitative RT-PCR.

Target gene	Primer	Nucleotide sequence
TNF-α	F	5’-CCCTCACACTCAGATCATCTTCT-3’
	R	5’-GCTACGACGTGGGCTACAG-3’
GAPDH	F	5’-ACCACAGTCCATGCCATCAC-3’
	R	5’-TCCACCACCCTGTTGCTGTA-3’

### Determination of NADPH oxidase activity

NADPH oxidase dependent ROS production was determined as previously described [29]. Briefly, RAW 264.7 macrophages cells were seeded in 96-well white culture plates at a density of 5*10^4^ cells/well. After overnight incubation, cells were treated with gAcrp for 24 hours and then lysed with HBSS containing 0.1% Triton X-100 and 0.1 N NaOH. Whole cellular lysates were then incubated with NADPH (200 mM) and lucigenin (100 mM) in HBSS at 37°C in the dark for 30 minutes. Chemiluminescence was recorded in relative light units every 5 minutes over a period of 60 minutes.

### Measurement of Total ROS production

Intracellular ROS production was measured essentially the same as previously described [29]. In brief, RAW 264.7 macrophages were seeded at a density of 5*10^4^ cells/well in 96-well black plate. Cells were treated with indicated concentration of gAcrp, and then treated with 10 μM CM-H_2_DCFDA in HBSS in dark for 30 minutes, followed by washing with HBSS. ROS production was measured by the change in fluorescence intensity immediately observed by fluorescence microscopy (Nikon, Tokyo, Japan).The fluorescence intensities were analyzed by quantitative digital analysis using Image Inside from FOCUS (Seoul, South Korea).

### Transient transfection with small interfering RNAs

RAW 264.7 macrophages were seeded on 35-mm or 60-mm dishes at a density of 7*10^5^ or 1*10^6^ cells/well respectively. After overnight incubation, cells were transfected with siRNA targeting LC3B (25 nM), FoxO3A (25 nM), SIRT1 (50 nM) or scrambled control siRNA using Hiperfect transfection reagent (Qiagen) according to the manufacturer’s instructions. Gene silencing efficiency was assessed by Western blot analysis after 24–48 hours of transfection. The siRNA duplexes were synthesized by Bioneer (Daejeon, South Korea) and sequences for the siRNA used are shown in Table 2.

**Table 2:** Sequences of small interfering RNA used in transfection.

Target gene	Primer	Nucleotide sequence
LC3B	F	5’-GUGGUUGUCAAGUGGUAGA-3’
	R	5’-UCUACCACUUGACAACCAC-3’
FoxO3A	F	5’-GACGUCAUGAUGACCCAGU-3’
	R	5’-ACUGGGUCAUCAUGACGUC-3’
SIRT1	F	5’-GACCGUUUAAUGACUGGAU-3’
	R	5’-AUCCAGUCAUUAAACGGUC-3’

### Preparation of cellular extract and Western blot analysis

For the preparation of total protein, cytosolic and nuclear fraction, RAW 264.7 macrophage cells were seeded in 35-mm dishes at a density 1*10^6^ cells/well or 60 mm dish at a density of 3*10^6^ cells/ well, respectively. After 24 hours incubation, cells were treated with gAcrp as indicated time points. Total proteins were then extracted using RIPA lysis buffer containing Halt protease and phosphatase inhibitor single-use cocktail (Thermo Scientific, Waltham, MA). For cytosolic and nuclear fraction, cells were lysed using subcellular fractionation buffer (250 mM Sucrose, 20 mM HEPES pH 7.4, 10 mM KCl, 1.5 mM MgCl2, 1mM EDTA, 1 mM EGTA, 1 mM DTT, and halt protease inhibitor cocktail). The cellular lysates were homogenized through 25 G syringes 10–15 times followed by keeping on the ice for 20 minutes and then centrifuged at 3,000 rpm for 5 minutes at 4°C to separate nuclear pellet. The supernatant was centrifuged at 8,000 rpm for 20 minutes and supernatant was taken as cytosolic fractions. For the preparation of nuclear fraction, nuclear pellet was lysed in RIPA buffer and protein was extracted as same process as total protein extraction. For immunoblot analysis, 20–30 μg of solubilized proteins were loaded and resolved by 8–15% SDS-PAGE. The proteins were then transferred to PVDF membranes, blocked with 5% skim milk in phosphate-buffered saline/Tween 20 for 1 hour, incubated with the designated primary antibodies overnight at 4°C, washed and incubated with the secondary horseradish peroxidase (HRP)–labeled anti-rabbit/mouse antibody (1:4000). Primary antibodies were diluted at a ratio of 1:2000 (1:500 in case of Lamin-B1) in 3% Bovine Serum Albumin (BSA). Chemiluminescent images of the blots were finally captured using a Fujifilm LAS-4000 mini (Fujifilm, Tokyo, Japan). The membranes were then stripped and re-probed with desired antibodies (β-actin or laminB1) as a loading control.

### Preparation of conditioned media

Conditioned media was prepared by same methods as described previously [30]. In brief, RAW 264.7 macrophages were stimulated with 1 μg/ml of gAcrp for 8 hours, washed and then incubated for additional 12 or 24 hours in growth media without gAcrp. Supernatants were then collected as conditioned media (CM). After centrifugation for 5 minutes at 5,000 g, the supernatants were mixed with fresh media at 2:1 ratio and used for incubation of RAW 264.7 macrophages. Media collected from the cells treated with media alone in the absence of gAcrp was used as the control group.

### Confocal microscopic analysis

For confocal microscopic analysis, RAW 264.7 macrophages were seeded at a density 5*10^4^ cells/well in 8-well chamber slides. After overnight incubation, cells were transfected with enhanced green fluorescent protein (eGFP)-LC3 expression plasmid using Fugene HD transfection reagent (Promega, Madison, USA) as described previously [22]. After 48 hour of transfection, cells were treated with gAcrp for the indicated time periods. Cells were then fixed with 4% paraformaldehyde solution and confocal images were taken using an A1 Confocal Laser Microscope System (Nikon Corp., Tokyo, Japan). Autophagic puncta was quantitated from confocal images from triplicate experiments. The images were expressed as percentage of cells with GFP-LC3 dots obtained from at least 100 cells with Image Inside software version 2.32.

### Statistical analysis

Values were presented as mean ± S.E.M. from at least three separate experiments. Data were analyzed by one-way analysis of variance (ANOVA) and Tukey’s multiple comparison tests using GraphPad Prism software version 5.01 (La Jolla, CA). Differences between groups were considered to be significant at P < 0.05.

## Results

### Globular adiponectin causes autophagy induction in RAW 264.7 macrophages and murine peritoneal macrophages

**Fig 1:**
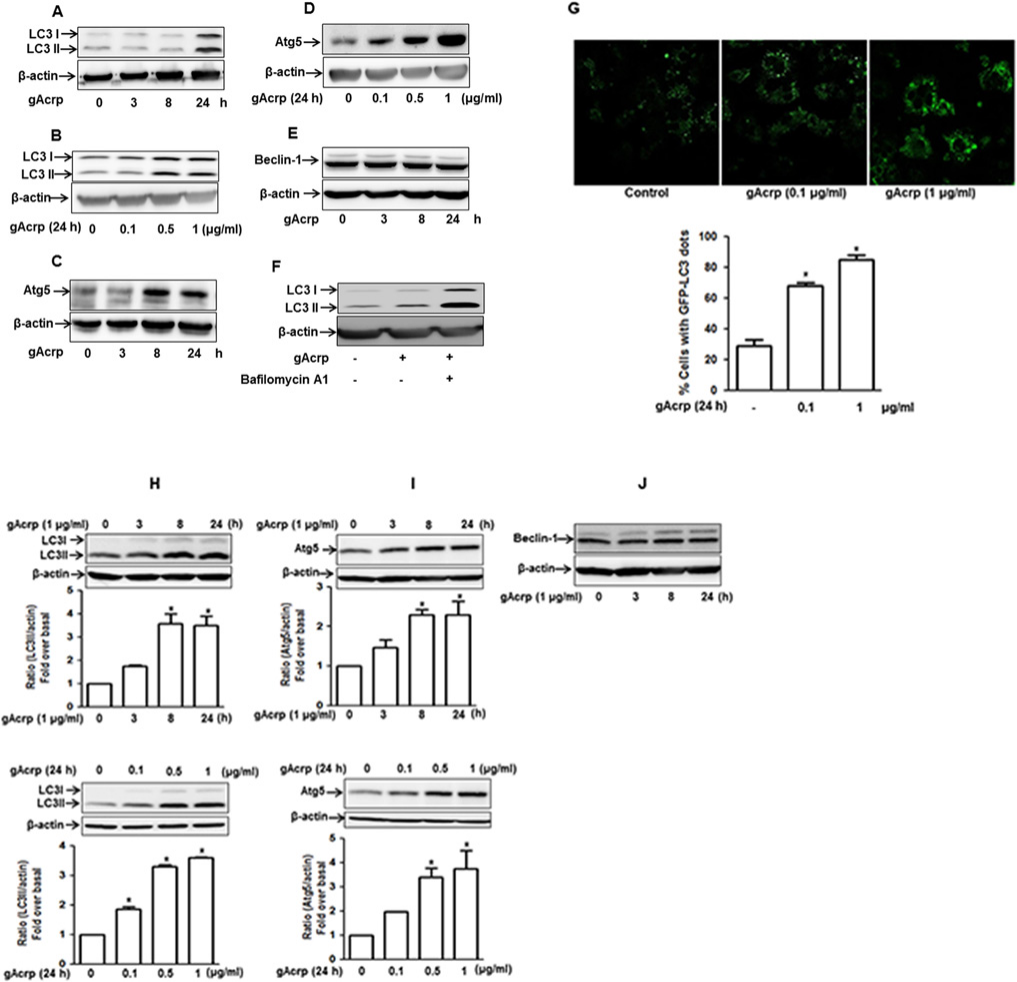
Effect of gAcrp on autophagy induction in RAW 264.7 macrophages and primary peritoneal macrophages. (A and B) RAW 264.7 macrophages were treated with gAcrp (1 μg/ml) for different time duration (A) or different concentrations for 24 h (B). LC3II expression level was determined by Western blot analysis as described in materials and methods. (C and D) Cells were treated with gAcrp (1 μg/ml) for the indicated time periods (C) or different concentrations for 24 h (D). Atg5 expression level was examined by Western blot analysis as described previously. (E) Cells were treated with gAcrp (1 μg/ml) for the indicated time periods. Beclin-1 expression level was determined by Western blot analysis. Representative image from three independent experiments has been shown along with β-actin as internal loading control. (F) Cells were pretreated with Bafilomycin A1 (10 nM) for 2 h, followed by treatment with gAcrp (1 μg/ml) for additional 24 h. LC3II protein level was examined by Western blot analysis as described previously. Images are representative of three independent experiments that showed similar results. (G) Cells were transiently transfected with eGFP-LC3 plasmid. After 48 h, cells were treated with indicated concentration of gAcrp for 24 h. GFP-LC3 dots formation was viewed with A1 Confocal Laser Microscope System as described in material and methods. Representative image from three independent experiments has been shown along with quantitation of LC3 dots (lower panel). Values are expressed as percentage of cells with GFP-LC3 dots obtained from at least 100 cells. (H, I and J) Primary peritoneal macrophages were isolated from mice as indicated in materials and methods. Treatment was done identical to those outlined in Fig 1A and B. Expression levels of LC3II (H), Atg5 (I) and Beclin-1 (J) were measured by Western blot analysis. Representative images are shown along with β-actin as internal loading control. Quantitative analysis of LC3II and Atg5 expression was performed by densitometric analysis and is shown in bar graph (lower panel). Values are presented as mean ± SEM. ^*^ *P* < 0.05 compared to control.

To investigate the role of autophagy in the development of tolerance to LPS-induced TNF-α expression by adiponectin, we first examined whether globular adiponectin (gAcrp) induces autophagy in RAW 264.7 macrophages. For this, we examined the effects of gAcrp on the expression of genes related with autophagy. As shown in Fig 1, treatment with gAcrp induced significant increase in expression of microtubule-associated protein light chain 3 (LC3), regarded as a major constituent of autophagosome (Fig 1A and 1B) and Atg5, which is necessary for the elongation of autophagosome (Fig 1C and 1D), in a time- and dose-dependent manner. However, gAcrp did not produce significant effect on the expression of Beclin-1, essential for vesicle nucleation (Fig 1E). In addition, treatment of cells with bafilomycin A1, an inhibitor of lysosomal machinery that blocks degradation of autophagosomal contents, further enhanced gAcrp-induced LC3 II protein expression (Fig 1F), suggesting that gAcrp induces autophagic flux in RAW 264.7 macrophages. Finally, autophagy inducing effect of gAcrp was further confirmed by analysis of autophagosome formation. In these experiments, cells were transfected with eGFP-LC3 plasmid, in which autophagosome formation correlates with the formation of LC3II-positive puncta viewed as aggregate dot-like structures. As expected, stimulation with gAcrp caused significant increase in number of LC3 puncta in a concentration dependent manner (Fig 1G). To further verify that gAcrp modulates expression of genes related with autophagy in other primary cells, we isolated macrophages from peritoneum of mice and explored the effects of gAcrp on the expression of autophagy markers. As depicted in Fig 1H and 1I, treatment with gAcrp significantly increased protein expression of LC3II (Fig 1H) and Atg5 (Fig 1I) in a time- and dose- dependent manner; whereas no significant effect was observed in Beclin-1 expression (Fig 1J), which were essentially similar to the results from RAW 264.7 macrophages.

### Globular adiponectin modulates expression of autophagy-related genes in a paracrine manner

**Fig 2:**
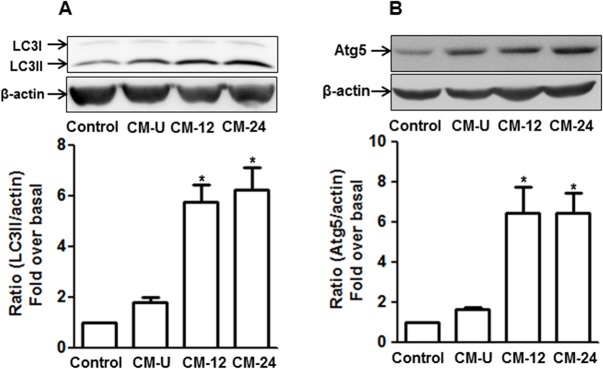
Paracrine effect of adiponectin on the expression of gene related to autophagy. Conditioned medium (CM) were prepared as described in materials and methods. After treatment with CM for 24 h, LC3II (A) and Atg5 (B) protein expression levels were determined by Western blot analysis. Images are representative of three independent experiments. Quantitative analyses of LC3II (A) and Atg5 (B) protein expression were performed by densitometric analysis and are shown in the graph (lower panel). Values are presented as mean ± SEM (n = 3). ^*^ *P* < 0.05 compared to control and CM-U. Control; Cells treated with DMEM containing 0.1% FBS, CM-U; CM prepared from gAcrp unstimulated cells, CM-12; Cells were stimulated with gAcrp for 8 h. After changing medium, cells were further incubated with 0.1% FBS/DMEM for 12 h without gAcrp and media was collected as CM-12, CM-24; Cells were stimulated with gAcrp for 8 h. After changing medium, cells were further incubated with 0.1% FBS/DMEM for 24 h without gAcrp and media was collected as CM-24.

Adiponectin predominantly produced in adipocytes is secreted to generate biological responses in target tissues and has been shown to act in a paracrine-dependent manner [30]. To further characterize the effects of gAcrp on autophagy induction, we examined the paracrine effect of gAcrp on the expression of genes related with autophagy. We prepared conditioned media from RAW 264.7 macrophages stimulated with gAcrp and analyzed its effect on the expression of LC3II and Atg5. As shown in Fig 2, treatment of cells with conditioned media induced significant increase in expression of LC3II (Fig 2A) and Atg5 (Fig 2B) compared with control and treatment with the media from unstimulated cells, suggesting that paracrine effect is involved in gAcrp-induced expression of LC3II and Atg5.

### Autophagy induction plays a role in the suppression of LPS-induced TNF-α expression by globular adiponectin in RAW 264.7 macrophages and murine peritoneal macrophages

**Fig 3:**
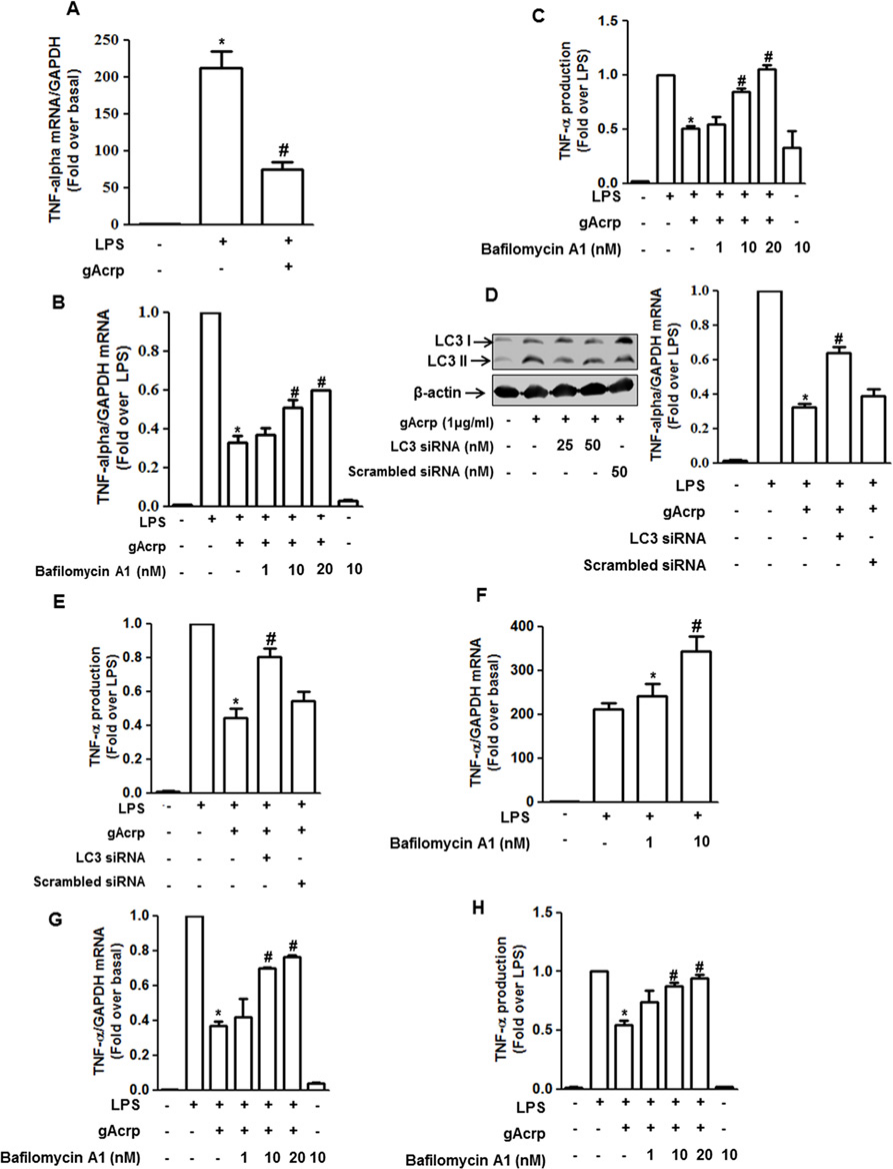
Role of autophagy in suppression of LPS-induced TNF-α expression by gAcrp in RAW 264.7 macrophages and peritoneal macrophages. (A) Cells were pretreated with gAcrp (1 μg/ml) for 24 h, followed by treatment with LPS (100 ng/ml) for additional 2 h. TNF-α mRNA level was assessed by qRT-PCR as described in materials and methods and normalized to GAPDH mRNA. Values represent fold increase compared with control cells and are expressed as mean ± S.E.M (n = 6). ^*^ *P* < 0.05 compared with untreated cell; ^#^ *P* < 0.05 compared to cells treated with LPS. (B) Cells were pretreated with Bafilomycin A1 (a selective autophagosome-lysosome inhibitor) for 2 h in the absence or presence of gAcrp (1 μg/ml) followed by stimulation with LPS (100 ng/ml) for additional 2 h. TNF-α mRNA expression was measured by qRT-PCR as described previously. Values represent fold change relative to LPS-treated cells and are presented as mean ± S.E.M. (n = 7). ^*^ *P* < 0.05 compared to cells treated with LPS; ^#^ *P* < 0.05 compared to cells treated with LPS and gAcrp. (C) Cells were pretreated with Bafilomycin A1 in the absence or presence of gAcrp (1 μg/ml) followed by stimulation with LPS (100 ng/ml) for additional 4 h. Secretion of TNF- α protein in the media was measured by ELISA as indicated in materials and methods. Values are presented as mean ± SEM. (n = 3). ^*^ *P* < 0.05 compared to cells treated with LPS; ^#^ *P* < 0.05 compared to cells treated with LPS and gAcrp. (D) (Left panel) Cells were transfected with indicated concentration of siRNA targeting LC3B or scrambled control siRNA as described in materials and methods. After 48 hours, LC3II protein expression level was determined by Western blot analysis to monitor the efficiency of gene silencing. (Right panel) Cells were transfected with siRNA targeting LC3B (25 nM) or scrambled control siRNA. After 24 h, cells were pretreated with gAcrp (1 μg/ml) for 24 h, followed by LPS treatment (100 ng/ml) for additional 2 h. TNF-α mRNA level was assessed by qRT-PCR. Values represent fold change relative to LPS-treated cells and are expressed as mean ± S.E.M. (n = 3). ^*^ *P* < 0.05 compared to cells treated with LPS; ^#^ *P* < 0.05 compared to cells treated with LPS and gAcrp. **(E)** Cells were transfected with siRNA targeting LC3B (25 nM) or scrambled control siRNA. After 24 h, cells were pretreated with gAcrp (1 μg/ml) for 24 h, followed by LPS treatment (100 ng/ml) for 4 h by changing the media. Secretion of TNF-α protein in the media was measured by ELISA. Values are presented as mean ± SEM. (n = 6). ^*^ *P* < 0.05 compared to cells treated with LPS; ^#^ *P* < 0.05 compared to cells treated with LPS and gAcrp. (F) Cells were pretreated with Bafilomycin A1 for 2 h, followed by treatment with LPS (100 ng/ml) for additional 2 h. TNF-α mRNA level was measured by qRT-PCR. Values represent fold change compared with control cells and expressed as mean ± S.E.M. (n = 5). ^*^ *P* < 0.05 compared to the control cells; ^#^ *P* < 0.05 compared to cells treated with LPS. (G and H) Macrophages were isolated from peritoneum of mice as described previously. Cells were pretreated with Bafilomycin A1 in the absence or presence of gAcrp and LPS essentially for the treatment of RAW 264.7 macrophages (shown in Fig 3B and C). TNF-α mRNA level was measured by RT-PCR and normalized to GAPDH mRNA (G) and the amount of TNF-α protein secreted to the media was measured by ELISA (H). Values are presented as mean ± S.E.M. (n = 3). ^*^ *P* < 0.05 compared with LPS; ^#^ *P* < 0.05 compared to cells treated with LPS and gAcrp.

Adiponectin has been shown to possess potent anti-inflammatory properties, particularly suppressing LPS-induced TNF-α expression [31]. Before investigating the role of autophagy in the suppression of TNF-α expression by gAcrp, we first confirmed the suppressive effect of gAcrp on LPS-induced TNF-α expression in our experimental condition. As shown in Fig 3A, pretreatment with gAcrp prevented LPS-induced TNF-α mRNA expression in RAW 264.7 macrophages consistent with the previous reports. Interestingly, pretreatment with Bafilomycin A1 significantly restored the suppression of LPS-induced TNF-α mRNA expression (Fig 3B) and secretion into media (Fig 3C) by gAcrp in RAW 264.7 macrophages. Pretreatment with Bafilomycin A1produced similar effects on both TNF-α mRNA expression and secretion in primary peritoneal macrophages (Fig 3G and 3H, respectively). Furthermore, knocking down of LC3B gene by siRNA transfection also significantly reversed the suppression of TNF-α mRNA expression (Fig 3D) and TNF-α protein secretion (Fig 3E) by gAcrp in RAW 264.7 macrophages. These findings suggest that suppressive effect of gAcrp on LPS-induced TNF-α expression is mediated, at least in part, via autophagy induction. Although the results from Fig 3A–3E suggested the role of autophagy induction in the suppression of LPS-induced TNF-α expression, in fact, LPS itself has been also shown to induce autophagy [32]. To address this discrepancy, we further explored the role of autophagy in LPS-induced TNF-α expression. As depicted in Fig 3F, LPS-induced TNF-α mRNA expression was further significantly enhanced by pretreatment with Bafilomycin A1, implying that autophagy plays an inhibitory role in TNF-α expression in macrophages treated with LPS.

### Autophagy induction is involved in the suppression of LPS-induced p38 MAPK phosphorylation and TRAF6 expression by gAcrp in RAW 264.7 macrophages

**Fig 4:**
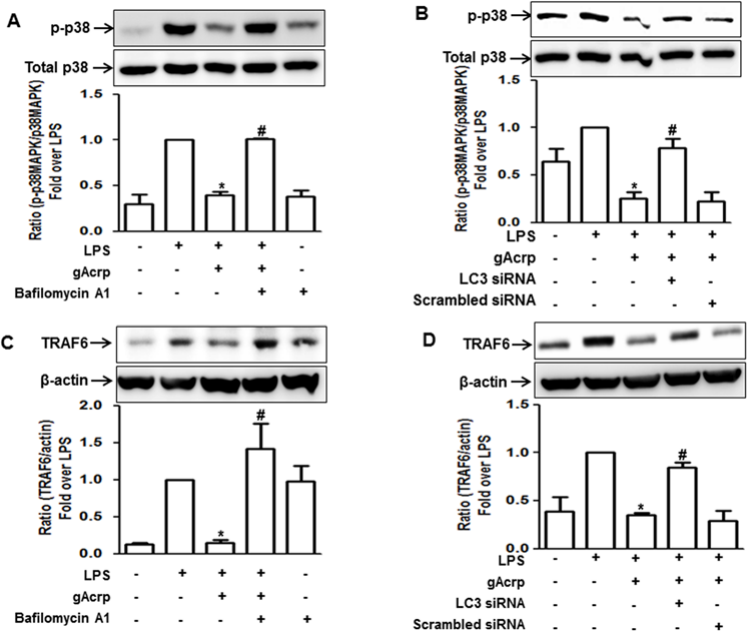
Role of autophagy induction in suppression of LPS-induced TRAF6 expression and p38MAPK phosphorylation by gAcrp. (A and C) Cells were pretreated with Bafilomycin A1 (10 nM) for 2 h followed by treatment with gAcrp (1 μg/ml) for additional 24 h. Cells were then treated with LPS (100 ng/ml) for 30 minutes. (B and D) Cells were transfected with siRNA targeting LC3B (25 nM) or scrambled control siRNA. After 48 hours, cells were treated with gAcrp (1 μg/ml) for 24 h, followed by treatment with LPS (100 ng/ml) for additional 30 minutes. Western blot analysis was performed to detect the level of phosphorylated p38MAPK (A, B) along with total p38MAPK, and TRAF6 (C, D) expression along with β-actin as an internal control. Representative images of three independent experiments that showed similar results are shown. Quantitative analysis of phosphorylated p38MAPK and TRAF6 expression were performed by densitometric analysis and are shown in the graph (lower panel of each figure). Values are presented as mean ± S.E.M. (n = 3).^*^ *P* < 0.05 compared with LPS treated cell; ^#^ *P* < 0.05 compared to cells treated with LPS and gAcrp.

To further identify the molecular mechanisms underlying suppression of TNF-α expression, we investigated the role of autophagy induction in the modulation of p38 MAPK phosphorylation and TRAF6 expression, since these are well known signaling events required for TNF-α expression, and further have been considered as targets for autophagic process [33,34]. For this, cells were pretreated with Bafilomycin A1 followed by LPS treatment in the presence of gAcrp. As shown in Fig 4, pretreatment with Bafilomycin A1 reversed the suppression of p38MAPK phosphorylation (Fig 4A) and TRAF6 expression (Fig 4C) by gAcrp, indicating a critical role of autophagy in the modulation of TRAF and p38MAPK by gAcrp. These results were confirmed by LC3B gene silencing. Knocking down of LC3B gene expression also caused restoration of the suppressive effects of gAcrp on p38MAPK phosphorylation (Fig 4B) and TRAF6 expression (Fig 4D), similar to the effects from Bafilomycin A1 pretreatment. All these data imply that autophagy induction plays an important role in the suppression of LPS-induced activation of TLR4 signaling in macrophages, particularly targeting p38MAPK and TRAF6.

### FoxO3A signaling is involved in gAcrp-induced expression of genes related with autophagy in RAW 264.7 macrophages

**Fig 5:**
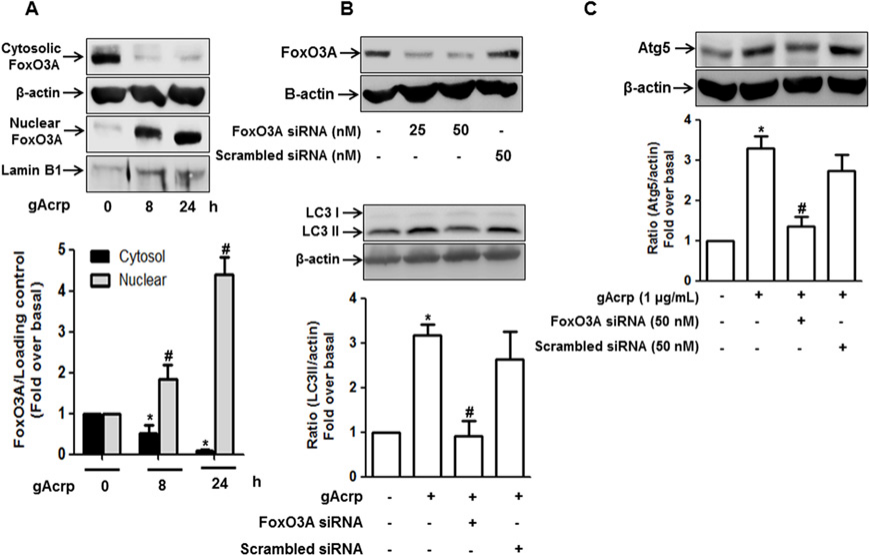
Role of FoxO3A signaling in the expression of autophagy-related genes by gAcrp in RAW 264.7 macrophages. (A) Cells were treated with gAcrp (1 μg/ml) for the indicated time periods. Cytosolic and nuclear protein fractions were prepared as described in the materials and methods and the level of FoxO3A in each fraction was determined by Western blot analysis along with β-actin and lamin B1 as internal loading control for cytosolic and nuclear fractions, respectively. Quantitative analyses of foxO3A in cytosol and nucleus were performed by densitometric analysis and are shown in the graph (lower panel). Values are presented as mean ± S.E.M. ^*^ *P* < 0.05 compared with control cells in the cytosol fraction, ^#^ *P* < 0.05 compared with control cells in the nucleus fraction, respectively. (B) and (C) Cells were transfected with indicated concentration of FoxO3A siRNA or scrambled control siRNA for 48 h. The efficiency of FoxO3A silencing was measured by Western blot analysis, keeping β-actin as an internal loading control (Upper panel of figure B). Cells were transfected with siRNA targeting FoxO3A (50 nM) or scrambled control siRNA. After 48 h incubation, cells were treated with gAcrp (1 μg/ml) for 24 h and LC3II (B) or Atg5 (C) protein expression levels were determined by Western blot analysis. Representative images from three independent experiments are shown keeping β-actin as an internal loading control. Quantitative analysis of LC3II and Atg5 expression were performed by densitometric analysis and are shown in the graph (lower panel of each figure). Values are presented as mean ± S.E.M. (n = 3). ^*^ *P* < 0.05 compared with control; ^#^ *P* < 0.05 compared to cells treated with gAcrp.

In a continuation study for investigation of mechanisms underlying autophagy induction by gAcrp in macrophages, we have examined the involvement of FoxO3A in gAcrp-induced autophagy activation, since nuclear translocation of FoxO3A is closely associated with transcriptional activation of genes related with autophagy [22]. For this, we first examined subcellular localization of FoxO3A in response to gAcrp treatment and observed that gAcrp treatment decreased the level of cytosolic FoxO3A (Fig 5A, upper panel), whereas nuclear accumulation was increased (Fig 5A, lower panel). Additionally, gene silencing of FoxO3A inhibited gAcrp-induced expression of LC3II (Fig 5B) and Atg5 (Fig 5C). All these data indicate that FoxO3A signaling plays a critical role in the expression of genes related with autophagy by gAcrp in macrophages.

### ROS production contributes to gAcrp-induced autophagy via modulation of FoxO3A in RAW 264.7 macrophages

**Fig 6:**
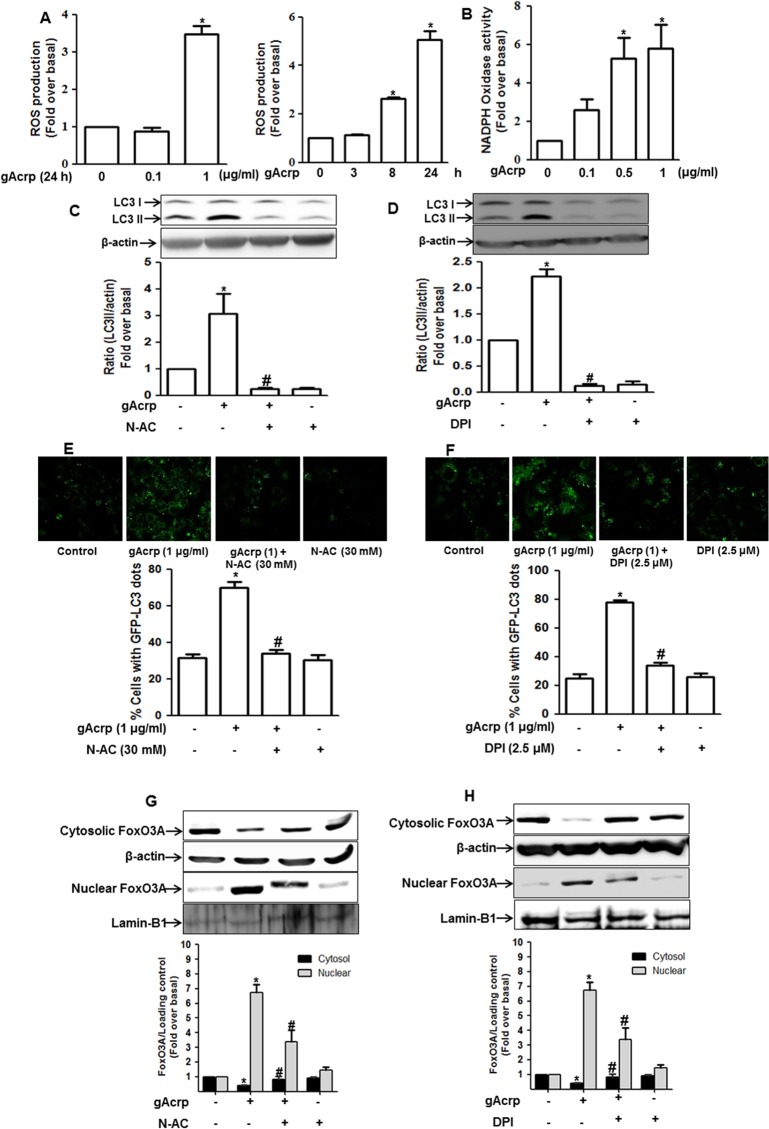
Role of ROS production in gAcrp-induced FoxO3A nuclear translocation and autophagy induction in RAW 264.7 macrophages. (A) Cells cultured in 96-well black plate were treated with different concentration of gAcrp for 24 h (left panel) or 1 μg/ml of gAcrp for different time duration (right panel). ROS production was determined using fluorometer as described previously. Data represent fold change compared to control cells and are expressed as mean ± SEM (n = 5), *P < 0.05 compared with control. (B) RAW 264.7 macrophages were treated with different concentration of gAcrp for 24 h. NADPH oxidase activity was determined by lucigenin-based assay as described in materials and methods. Values represent fold increase in compared to control cells and are expressed as mean ± S.E.M. (n = 3). *P < 0.05 compared with control cells. (C and D) Cells were pretreated with N-AC (30 mM) (C) or DPI (2.5 μM) (D) for 1 h, followed by treatment with gAcrp (1 μg/ml) for additional 24 h. LC3II protein expression level was measured by Western blot analysis as described previously. Images are representative of three independent experiments along with β-actin as internal loading control. LC3II protein expression was quantitated by densitometric analysis and is shown in the graph (lower panel) and values are presented as mean ± S.E.M. (n = 3). ^*^ *P* < 0.05 compared with control; ^#^ *P* < 0.05 compared to cells treated with gAcrp. (E and F) Cells were transiently transfected with eGFP-LC3 plasmid. After 48 h incubation, cells were pretreated with N-AC (E) or DPI (F) for 1 h followed by treatment with gAcrp for additional 24 h. GFP-LC3 dots formation was viewed with A1 Confocal Laser Microscope System as described previously. Representative images from three independent experiments that showed similar results are shown along with quantitation of LC3 dots (lower panel). Values are expressed as percentage of cells with GFP-LC3 dots obtained from at least 100 cells. ^*^ *P* < 0.05 compared with control; ^#^ *P* < 0.05 compared to cells treated with gAcrp. (G and H) Cells were pretreated with N-AC (G) or DPI (G) for 1 h followed by treatment with gAcrp (1 μg/ml) for additional 24 h. Cytosolic and nuclear protein fractions were prepared as described previously and the expression level of FoxO3A in each fraction was determined by Western blot analysis. Images are representative of three separate experiments that showed similar results along with β-actin and laminB1 as internal loading control for each fraction. Densitometric analysis was performed to quantitate protein and is shown in the graph (lower panel) and values are presented as mean ± S.E.M. (n = 2–3). ^*^ *P* < 0.05 compared with control; ^#^ *P* < 0.05 compared to cells treated with gAcrp in the cytosol and nucleus fraction.

Oxidative stress has been considered one of the most critical factors to induce autophagy [35]. To further elucidate the molecular mechanisms for gAcrp-induced autophagy induction, we assessed the potential role of ROS production in autophagy induction by gAcrp in RAW 264.7 macrophages. As shown in Fig 6A, gAcrp treatment increased ROS production in a dose (left panel) and time (right panel) dependent manner. In these experiments, relatively high concentration of gAcrp (1 μg/ml) was required for ROS production, but low concentration (0.1 μg/ml) was not enough to induce, which is consistent with the previous report [29,36]. In addition, gAcrp treatment increased NADPH oxidase activity, assessed by lucigenin assay, in a dose dependent manner (Fig 6B), implying that gAcrp increases ROS production via modulation of NADPH oxidase. Finally, to verify the functional role of ROS production in gAcrp-induced autophagy, cells were pretreated with N-AC (ROS scavenger) or DPI (NADPH oxidase inhibitor) and LC3II protein expression level and autophagosome formation was measured. We observed that pretreatment with N-AC and DPI prevented gAcrp-induced LC3II protein expression (Fig 6C and 6D), as well as blocked gAcrp-induced increase in autophagosome formation (Fig 6E and 6F). All these results indicate the critical role of ROS production in autophagy activation by gAcrp. Recent studies have also demonstrated that FoxO3A is activated in ROS-dependent manner [20]. We therefore further examined whether ROS production is involved in the activation of FoxO3A by gAcrp in RAW 264.7 macrophages. As shown in Fig 6A, gAcrp-induced nuclear translocation of FoxO3A was significantly prevented by pretreatment with N-AC (Fig 6G) and DPI (Fig 6H), whereas cytosolic level was enhanced, indicating an important role of ROS production in activation of FoxO3A in response to gAcrp and further autophagy induction in RAW 264.7 macrophages.

### SIRT1 is involved in gAcrp-induced FoxO3A activation in RAW 264.7 macrophages

**Fig 7:**
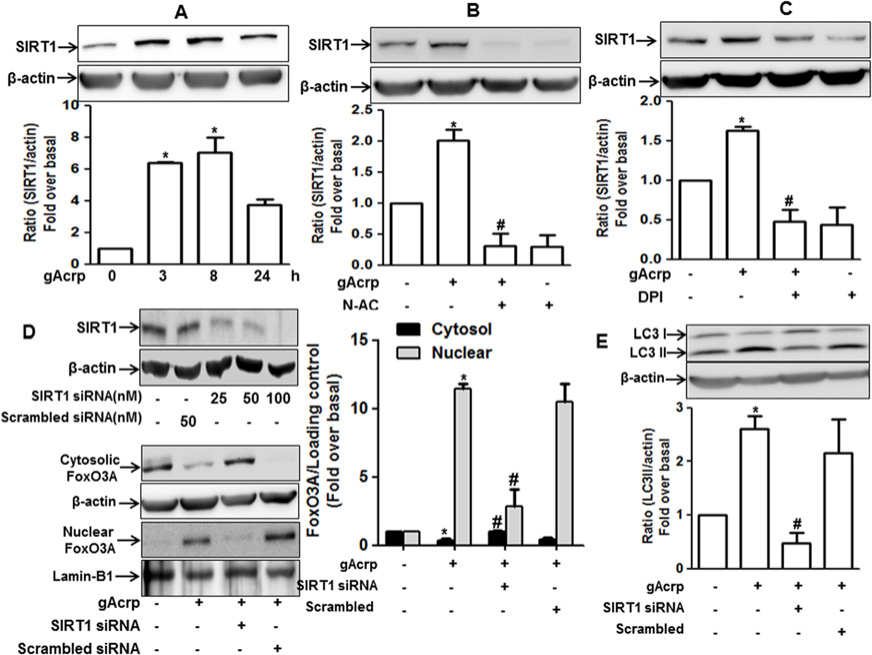
Role of SIRT1 in gAcrp-induced FoxO3A nuclear translocation and autophagy induction in RAW 264.7 macrophages. (A) Cells were treated with gAcrp (1 μg/ml) for indicated time periods. SIRT1 protein expression was determined by Western blot analysis as described previously. (B and C) Cells were pretreated with N-AC (B) or DPI (C) for 1 h followed by treatment with gAcrp (1 μg/ml) for additional 8 h. Western blot analysis was performed to determine SIRT1 protein expression level. Representative images of three independent experiments are shown, keeping β-actin as an internal loading control. Quantitative analysis for SIRT1 expression was performed by densitometric analysis and presented as mean ± S.E.M. (n = 3). ^*^ *P* < 0.05 compared with control; ^#^ *P* < 0.05 compared to cells treated with gAcrp. (D) (Upper panel) Cells were transfected with indicated concentration of siRNA targeting SIRT1 or scrambled control siRNA. Gene silencing efficiency was measured by Western blot analysis. (Lower panel) After transfection with SIRT1 siRNA or scrambled siRNA, cells were treated with gAcrp (1 μg/ml) for 24 h. FoxO3A protein expression levels in cytosol and nucleus were determined by Western blot analysis along with β-actin and lamin B1 as internal loading control for cytosolic and nuclear respectively. Images are representative of three independent experiments. Densitometric analysis was done to quantitate protein and is shown in the graph (right panel) and values are presented as mean ± S.E.M. (n = 3). ^*^ *P* < 0.05 compared with control; ^#^ *P* < 0.05 compared to cells treated with gAcrp in the cytosol and nucleus fraction. (E) RAW 264.7 macrophages were treated with SIRT1 siRNA and gAcrp essentially same as above. LC3II expression level was monitored by Western blot analysis as described previously. Quantitative analysis for FoxO3A and LC3II protein expression were performed by densitometric analysis and are shown in the graph (lower panel of each figure). Values are expressed as mean ± SEM (n = 3). ^*^ *P* < 0.05 compared with control; ^#^ *P* < 0.05 compared to cells treated with gAcrp.

Oxidative stress is known to induce expression of SIRT1, acting as a deacetylase of various non-histone, as well as histone substrates. In addition, deacetylation of FoxO3A causes nuclear translocation [37,38]. We next therefore investigated the potential role of SIRT1 in mediating FoxO3A activation by gAcrp. We found that gAcrp significantly increased expression of SIRT1 protein (Fig 7A), which was abolished by pretreatment with N-AC and DPI (Fig 7B and 7C), suggesting that gAcrp induces increase in SIRT1 expression via ROS-dependent manner. Furthermore, silencing of SIRT1 expression prevented gAcrp-induced nuclear translocation of FoxO3A (Fig 7D) and LC3II protein expression (Fig 7E), implying that SIRT1 expression contributes to activation of FoxO3A and further subsequent expression of genes related with autophagy by gAcrp in RAW 264.7 macrophages.

## Discussion

Inflammation, a complex physiological response to the stimuli, is associated with various pathophysiological conditions, while it is also required for the maintenance of normal physiology. Inflammatory response is balanced by a number of pro- and anti-inflammatory mediators. Among them, Tumor Necrosis Factor-alpha (TNF-α) secretion by endotoxin has been considered as a critical event leading to the development of various diseases associated with acute and chronic inflammation [39]. Therefore, modulation of TNF-α production would be an effective strategy for the management of inflammation-related diseases. It is widely known that adiponectin, an adipokine predominantly secreted from adipose tissue, causes tolerance to LPS-induced TNF-α production. However, its mechanisms are not clearly understood. For the elucidation of the molecular mechanisms underlying, herein, we provided the first evidence that globular adiponectin suppressed TNF-α production, at least in part, via autophagy induction.

Adiponectin has been well known as a potent anti-inflammatory molecule. For example, adiponectin generates complex effects on toll-like system 4 (TLR4)-dependent signal transduction by shifting macrophages from classically activated phenotype (M1) to alternatively activated anti-inflammatory phenotype (M2) [4,40]. In addition, adiponectin inhibits TNF-α production via multiple mechanisms acting both at transcriptional and post-transcriptional level [31,41] in response to LPS stimulation in macrophages. For the regulation of TNF-α production, adiponectin has been shown to suppress LPS-stimulated transcriptional activity of NF-κB and phosphorylation of p38MAPK [42]. However, based on our understanding, the molecular mechanisms underlying suppression of LPS-induced TNF-α expression by adiponectin are still very limited. In the present study, we have demonstrated that suppressive effect of globular adiponectin on LPS-induced TNF-α expression is mediated, at least in part, via autophagy induction. Furthermore, this effect was due to the suppression of LPS-induced TRAF6 expression and p38MAPK phosphorylation. In a complex of TLR4 signal transduction, p38MAPK is a downstream target molecule of TRAF6 via MKK activation [43]. Therefore, it seems sequestration of TRAF6 by autophagosome may cause the inhibition of p38MAPK phosphorylation and then TNF-α expression. In addition, autophagy induction by gAcrp was dependent on ROS-mediated SIRT1 expression and FoxO3A nuclear translocation.

In addition to the critical role in determination of cell death and/or survival [44], recent studies have demonstrated that autophagic process is implicated in diverse biological responses. In particular, autophagy has been shown to play a critical role in the regulation of inflammatory responses. For example, the induction of autophagy has been found to be aligned with attenuation of LPS-induced inflammation [45] and inhibition of autophagy contributes to the augmentation of TNF-α and IL-1β expression in LPS-stimulated intestinal epithelium [46], suggesting the critical role of autophagy in suppressing inflammatory responses. In addition, autophagy plays a cytoprotective role in LPS-induced acute kidney disease [47]. In agreement with these reports, autophagy induction also modulates the molecular components in TLR4 signaling. For example, autophagy induction can suppress LPS-induced TRAF6 expression [34], while inhibition of autophagy signifies the inflammation and cytotoxicity by augmenting IKK, p38 and JNK activation [48,49]. We and others have recently demonstrated that adiponectin induces autophagy, ensuring the protection of cells from ethanol-induced liver injury and glucose deprivation [22,50]. Therefore, it is widely accepted that autophagy plays a role in the regulation of inflammatory responses and adiponectin induces autophagy. However, the potential involvement of autophagy in the modulation of LPS-stimulated production of inflammatory cytokines by adiponectin has not been reported. In the present study, we found that autophagy induction plays a critical role in the suppression of TNF-α expression (Fig 3) via attenuation of LPS-induced TLR4 signaling events in RAW 264.7 macrophages (Fig 4). To the best of our knowledge, this is the first report to show the potential role of autophagy in the regulation of pro-inflammatory cytokines production by adiponectin in macrophages.

Autophagic process is composed of multiple steps, including vesicle nucleation, elongation, closure of autophagosome and finally fusion with lysosome. Each of this process is coordinately regulated by a number of genes related with autophagy. In mammals, autophagy induction is usually initiated by inhibition of mTOR by Ulk1 (Unc-51-like kinase) and Ulk2. The vesicle nucleation is governed by formation of complex of Beclin-1 (BECN1) with class III PI3K (phosphatidylinositol-3 kinase) along with Atg14. Further, two protein conjugation systems are involved in elongation of vesicle. One is complexation of ATG16L1 with Atg5-12 conjugate and the other is lipidation of LC3I with PE (phosphatidyl ethanolamine) leading to LC3II formation. Autophagosome formation is completed by LC3II and matured by fusion with lysosome [51]. Due to the localization of lipidated form of LC3 to autophagosome membrane, LC3II is usually regarded as a marker for autophagy induction and autophagosome formation [11]. In the present study, we found that gAcrp increased the expression of Atg5 and LC3II (Fig 1A–1D), while it did not affect Beclin-1 expression in RAW 264.7 macrophages (Fig 1E), implying that gAcrp induced non-canonical Beclin-1 independent autophagy. In fact, in many cases, autophagy process is induced by Beclin-1 independent manner. For example, Seo and colleagues have reported that H_2_O_2_ induces autophagy by degradation of Rheb (an upstream activator of mTOR) in Beclin-1 independent way in GSH-depleted RAW 264.7 cells [52]. In addition, ZnPPIX induces autophagy through p38MAPK activation without involvement of Beclin-1 [53]. These reports are different from the conventional autophagy pathway including Beclin-1. Based on these reports and the results from the present study, we concluded that globular adiponectin induces autophagy in RAW 264.7 macrophages via Beclin-1-independent non-canonical pathway.

In the present study, we suggested that adiponectin treatment suppressed LPS-induced TNF-α expression via autophagy induction. However, previous studies have also reported that LPS treatment itself promotes autophagy induction in RAW 264.7 macrophages [54], Herein, we have shown that inhibition of autophagy (pretreatment with bafilomycin A) further enhances LPS-induced TNF-α expression (Fig 3F), implying that autophagy induction plays an inhibitory regulatory role in TNF-α expression by LPS. Consistent with this finding, a recent study has reported that inhibition of autophagy by knocking down of Atg7 sensitizes Kupffer cells, liver resident macrophages, to LPS stimulation by augmenting the activation of p38 and IKK [48]. In contrast, other reports have demonstrated that LPS-induced autophagy correlates to the expression of inflammatory cytokines [32] and liver inflammation causing organ injury and then deterioration of liver function in diabetic rats [55]. Therefore, the role of autophagy in the production of inflammatory cytokines in LPS-stimulated macrophages is still controversial and would be context dependent. Detailed mechanistic understandings regarding what determines autophagy acts as a pro- or anti-inflammatory mediator in LPS-stimulated macrophages remains to be investigated.

FoxO3A is activated in response to various stressful stimuli and well known to induce expression of autophagy-related genes [56]. Consistent with previous reports, we found that gAcrp induced LC3II and Atg5 protein expression through FoxO3A dependent manner (Fig 5A–5C). The transcriptional up-regulation or activation of FoxO3A is governed by various stressful stimuli. In particular, oxidative stress has been considered as one of the critical factors to induce FoxO3A activation. For example, oxidative stress caused by Hydrogen peroxide (H_2_O_2_) promotes nuclear localization of FoxO3A along with increased expression of target genes, such as anti-oxidant, anti-apoptotic and autophagy-related genes [57]. It is also well known that ROS production contributes to autophagy induction in various experimental conditions. For example, NOX2-derived ROS plays a key role in LC3 recruitment to the phagosomes that contains bacteria and promotes its maturation for killing [58]. In this report, ROS triggers LC3 lipidation and association of endogenous LC3 and ATG12, which are critical steps in autophagy process. In addition, ROS has been shown to trigger autophagic cell death in macrophages via caspase-independent manner and is mediated by ADP-ribose polymerase (PARP) activation [59]. In contrast to this notion, ROS-induced autophagy induction has also been shown to contribute to cellular survival mediated by either FoxO3A signaling or by JNK activation [60–62]. Based on these findings, it seems that ROS plays a crucial role in autophagy induction, while subsequent biological responses induced by ROS production are determined by context-dependent manner. Herein, we also demonstrated that gAcrp caused nuclear translocation of FoxO3A and subsequent autophagosome formation in macrophages via ROS-dependent manner (Fig 5). In fact, adiponectin has been considered to possess anti-oxidant properties [29]. However, Akifusa and colleagues reported that gAcrp treatment caused ROS production, which leads to the apoptosis of macrophages [36,63]. We previously reported that low dose of gAcrp (0.1 μg/ml) was not effective on ROS production, while higher dose (above 1 μg/ml) significantly increased ROS production in macrophages [29]. These results were also observed in this study (Fig 6A and 6B), indicating that adiponectin generates differential role in ROS production depending on experimental conditions, although gAcrp treatment causes ROS production in the present study.

Localization of FoxO3A is determined by post-translational modifications. Of the various post-translational modifications, acetylation/deacetylation status is critical to determine the localization of FoxO3A. Acetylation causes export of FoxO3A to the cytosol, while deacetylation status of FoxO3A causes localization in the nucleus and induces transcription of target genes. For example, CREB binding protein (CBP) and p300, acting as histone acetyltransferases (HATs), and Sirt family proteins, known as deacetylases, have been proposed to modulate FoxO3A function [64]. SIRT1, acting as histone deacetylase (HDAC), has exhibited diverse biological properties, including anti-oxidant and anti-apoptotic effects [20]. Furthermore, adiponectin has been shown to utilize SIRT1 expression in many biological responses, including protection of liver from alcoholic/non-alcoholic damage and inhibition of apoptosis in pancreatic cancer cell [65,66]. Based on these previous reports, we hypothesized that adiponectin induces FoxO3A activation via ROS-dependent SIRT1 expression. In accordance with the previous observations, we clearly demonstrated that globular adiponectin increased SIRT1 expression via ROS dependent manner (Fig 7A–7C), which is required for nuclear translocation of FoxO3A and subsequent LC3II expression (Fig 7D and 7E).

**Fig 8:**
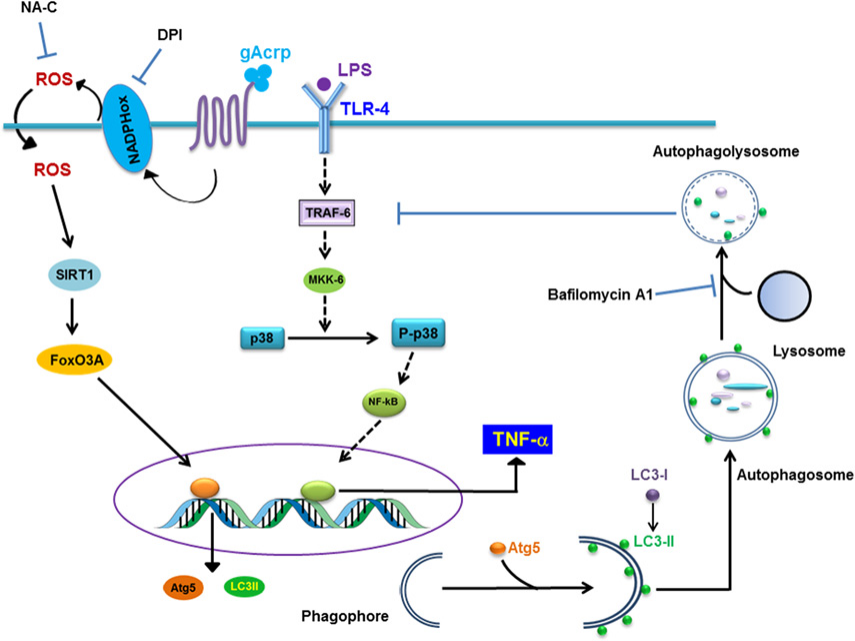
Proposed model of role of globular adiponectin-induced autophagy on suppression of LPS-induced TNF-α expression in RAW 264.7 macrophages. LPS treatment induces increase in TNF-α expression in macrophages via activation of TLR4 signaling, including TRAF6 induction, MKK-6 activation, p38MAPK phosphorylation and transcriptional activation of NF-κB. Treatment of macrophages with globular adiponectin causes expression of genes related with autophagy, including LC3II and Atg5, through activation of FoxO3A signaling, which is dependent on ROS production and SIRT1 expression. The autophagosome formation by gAcrp causes sequestration and degradation of TRAF6, further dampens the LPS-activated TLR4 signaling by inhibition of phosphorylation of p38MAPK, leading to the inhibition of LPS-stimulated TNF-α expression. Detailed molecular mechanism explaining how autophagy degrades TRAF6 and inhibits p38MAPK phosphorylation remained to be determined.

In conclusion, the data presented in this study demonstrates for the first time that autophagy induction contributes to the generation of tolerance to LPS-induced TNF-α expression by globular adiponectin in macrophages. Moreover, ROS/SIRT1/FoxO3A axis plays a crucial role in autophagy induction by globular adiponectin. Detailed molecular mechanisms underlying are illustrated in Fig 8. Based on these findings, we suggest that autophagy induction would be a novel mechanism underlying anti-inflammatory responses by adiponectin and SIRT1 and FoxO3A would be critical targets for the treatment of diseases associated with inflammation. Further studies are required to validate these findings from RAW 264.7 macrophages to the appropriate *in vivo* model.
