# Correction: Globular Adiponectin Causes Tolerance to LPS-Induced TNF-α Expression via Autophagy Induction in RAW 264.7 Macrophages: Involvement of SIRT1/FoxO3A Axis

**DOI:** 10.1371/journal.pone.0130370

**Published:** 2015-06-19

**Authors:** The PLOS ONE Staff

The first two authors, Nirmala Tilija Pun and Amit Subedimm, should be noted in the PDF version of the article as contributing equally to this work. The publisher apologizes for this error.
